# Invasively-treated incidence of lower extremity peripheral arterial disease and associated factors in Taiwan: 2000–2011 nationwide hospitalized data analysis

**DOI:** 10.1186/1471-2458-13-1107

**Published:** 2013-12-01

**Authors:** Nien-Tzu Chang, Chien-Lung Chan, Yu-Tzuen Lu, Jin-Chyr Hsu, Yuan-Nian Hsu, Dachen Chu, Nan-Ping Yang

**Affiliations:** 1 Department of Nursing, College of Medicine, National Taiwan University, Taipei, Taiwan; 2 Department of Information Management, Yuan-Ze University, Taoyuan, Taiwan; 3 Department of Medical Research, Taoyuan General Hospital, Ministry of Health & Welfare, Taoyuan, Taiwan; 4 Department of Surgery, Taipei-City Hospital, Taipei, Taiwan; 5 Institute of Public Health, National Yang-Ming University, Taipei, Taiwan

**Keywords:** Treated incidence, Associated factors, Lower extremity, Peripheral arterial disease

## Abstract

**Background:**

Lower extremity (LE) peripheral artery disease (PAD), which is associated with a reduced quality of life and increased mortality from atherosclerotic cardio-/cerebro-vascular occlusion, is a significant public health problem, especial for an aging society such as that of Taiwan.

**Methods:**

Specific datasets of the 2000–2011 nationwide inpatient databases were analyzed. Two inclusion criteria, including one of the major diagnosis codes of PAD and one of three categorical invasive treatments of LE PAD, were used consecutively to select cases diagnosed as LE PAD and receiving invasive treatment. The epidemiology of invasively-treated PAD in Taiwan was estimated, and the influences of potential confounders on these invasively-treated methods were evaluated.

**Results:**

In general, the invasively-treated incidence of PAD in Taiwan doubled, from 3.73/10,000 (in 2000) to 7.48/10,000 (in 2011). On average, the total direct medical cost of one hospitalized and invasively-treated PAD case ranged from $US 4,600 to $US 5,900. The annual cases of bypass surgery for the PAD cases averaged 1,000 and the cases for limb amputation ranged from 4,100 to 5,100 annually. However, the number of percutaneous transluminal angioplasty (PTA) procedures remarkably increased by 15 times, from 600/year to 9,100/year, from 2000 to 2011. 51.3% of all the enrolled cases were treated with limb amputations, and female, young and middle-aged people (30–65 years of age), DM patients and those on a low income had a tendency to undergo amputation due to PAD. 37.6% of all the enrolled cases were treated with PTAs related to hypertension, cardiovascular disease, hyperlipidemia and catastrophic Illness. 2-year PTA failure rates of 22.13%, 11.91% and 10.61% were noted among the first (2000–2001), second (2004–2005) and the third (2008–2009) cohort groups, respectively.

**Conclusions:**

In Taiwan, a gender difference and age and period effects on the invasively-treated incidence of LE PAD were observed. Female, young and middle-aged people (30–50 and 50–65 years of age), DM patients and those on a low income had a tendency to undergo amputation. The number of PTA procedures remarkably increased, but the 2-year failure rate of PTAs reduced from 2000 to 2011.

## Background

Peripheral artery disease (PAD) is a common condition used to describe the impairment of blood flow, especially to the lower extremities, that is usually a result of atherosclerotic occlusion. PAD occurrence has been noted to increase dramatically with age, and black race/ethnicity, current smoking, diabetes, hypertension, hypercholesterolemia, and low kidney function were found to be positively associated with prevalent PAD [1]. Otherwise, a family history of PAD is independently strongly associated with PAD prevalence and severity. This indicates a role of genetic factors or other shared environmental factors, or both, contributing to PAD [2].

Lower extremity (LE) PAD with clinical symptoms causes a significant reduction in quality of life [3]. More importantly, it is a marker of atherosclerotic disease burden, and is associated with increased mortality from cardiovascular and cerebrovascular causes [4]. A meta-analysis study including sixteen population cohort studies (a total of 480,325 person-years of follow-up of 24,955 men and 23,339 women) concluded that a low ankle-brachial index (ABI) (< or = 0.90) was associated with approximately twice the 10-year total mortality, cardiovascular mortality, and major coronary event rates as compared with the overall rate in each Framingham risk score (FRS) category [5].

However, approximately 40%-50% of patients with PAD are asymptomatic [4,6]; therefore, it is difficult to estimate its true prevalence in a community. Under-diagnosis and under-treatment of PAD are predicted in many countries. In patients with PAD, antiplatelet drugs and statins, exercise rehabilitation programs, lower extremity angioplasty, or bypass surgery are the current management strategies, and limb amputation is a critical method to save patients’ lives [7].

Therefore, PAD is a significant public health problem, especial for an aging society such as that of Taiwan. From the viewpoint of epidemiology of nationwide medical utilization, the present study aimed (1) to estimate the hospitalized incidence of PAD patients in Taiwan who were admitted to receive invasive treatments, and (2) to analyze the possible factors related to the different invasive treatment methods.

## Methods

### Source, security, and quality control of data

Taiwan launched a single-payer National Health Insurance (NHI) Program on March 1, 1995. As of 2011, 23.199 million of Taiwan’s total population of 23.225 million were enrolled in this program; foreigners in Taiwan are also eligible for inclusion. All enrollees enjoy almost free access to healthcare with a small co-payment by most clinics and hospitals [8]. This universal national health insurance, financed jointly by payroll taxes, subsidies, and individual premiums, commenced in Taiwan, and its coverage expanded from 57% of the population (before the introduction of national health insurance) to more than 98% (after the year 2005), and then to 99% (after the year 2007). The NHI has consistently received a 70 percent public satisfaction rate [9]. In order to respond to current and emerging health issues rapidly and effectively, the National Health Research Institute (NHRI), in cooperation with the National Health Insurance Bureau (NHIB), established a nationwide research database. The NHIB has established a uniform system to control the quality of medical services and coding. The NHRI safeguards the privacy and confidentiality of those included in the database and routinely transfers health insurance data from the NHIB to enable health researchers to analyze and improve the health of Taiwan’s citizens. The NHI database contains registration files and original claims data for reimbursement, and access to the National Health Insurance Research Database (NHIRD), which was derived from this system by the NHIB and is maintained by the NHRI, is provided to scientists in Taiwan for research purposes [10-13].

### Data protection and permission

Data in the NHIRD that could be used to identify patients or care providers, including medical institutions and physicians, are scrambled before being sent to the NHRI for database inclusion, and are further scrambled before being released to each researcher. Theoretically, it is impossible to query the data alone to identify individuals at any level using this database. All researchers who wish to use the NHIRD and its data subsets are required to sign a written agreement declaring that they have no intention of attempting to obtain information that could potentially violate the privacy of patients or care providers. This study protocol was evaluated by the NHRI, who gave their agreement to the planned analysis of the NHIRD (Agreement Number: NHIRD-101-566). This study was also approved by the Institutional Review Board (IRB) of Taoyuan General Hospital, which has been certificated by the Department of Health, Taiwan (IRB Approval Number: TYGH101049).

### Data selection and definitions of PAD, its related treatments and other co-morbidities

Specific datasets of the NHI research database, including “monthly claims summary for inpatient claims”, “inpatient cost by admission” and “details of inpatient orders” from between 2000 and 2011 were analyzed. In order to investigate the invasively-treated incidence of PAD, the International Classification of Diseases, 9th Revision, Clinical Modification (ICD-9-CM) diagnosis codes and ICD-9-CM treatment codes were evaluated. Two inclusion criteria were used consecutively to select cases that were diagnosed as PAD and received one of the invasive treatment methods for LE PAD. The major diagnosis codes of PAD and three categorical invasive treatments of LE PAD were defined as shown in Table 1. Otherwise, the potential risk factors of invasive treatments and patients’ demographic information, socioeconomic characteristics, and pre-existing diseases, cardiovascular disease, diabetes mellitus, end-stage renal disease (ESRD), hyperlipidemia, and severity of co-morbidities were included.

**Table 1:** ICD-9-CM definitions of PAD and related treatments

**Diagnoses or Treatments**	**Range of ICD-9-CM codes**
**Diagnoses: PAD**	**440.0 (atherosclerosis of aorta)**
	**440.2 (atherosclerosis of native arteries of the extremities),**
	**440.3 (atherosclerosis of bypass graft of the extremities),**
	**440.8 (atherosclerosis of other specific arteries)**
	**440.9 (other atherosclerosis of native arteries of the extremities)**
	**443 (other peripheral vascular disease)**
	**444.0 (arterial embolism, thrombosis of abdominal aorta)**
	**444.22 (arterial embolism & thrombosis of lower extremity)**
	**444.8 (arterial embolism, thrombosis of other specific artery)**
	**444.9 (embolism & thrombosis of unspecific artery)**
	**447.8 (other specific disorders of arteries and arterioles)**
	**447.9 (unspecific disorders of arteries and arterioles)**
**Treatments: PTA series (Percutaneous Transluminal Angioplasty)**	**38.08 (incision of vessel, lower limb arteries)**
	**38.18 (endarterectomy, lower limb arteries)**
	**38.38 (resection of vessel with anastomosis, lower limb arteries)**
	**38.48 (resection of vessel with replacement, lower limb arteries)**
	**38.68 (other excision of vessel, lower limb arteries)**
	**38.88 (other surgical occlusion of vessel, lower limb arteries)**
	**39.50 (angioplasty or atherectomy of non-coronary vessel)**
	**39.7 (Endovascular repair of vessel)**
	**39.90 (insertion of non-coronary artery stent)**
**Treatments: Bypass surgery**	**39.25 (aorta-iliac-femoral bypass)**
	**39.26 (other intra-abdominal vascular shunt or bypass)**
	**39.29 (other(peripheral) vascular shunt or bypass)**
**Treatments: Amputation**	**84.1 (amputation of lower limb)**
	***84.10 ~ 84.15 low level amputation (amputation below knee)***
	***84.16 ~ 84.19 high level amputation (knee disarticulation or above)***

If a patient’s ailment is diagnosed by a physician as a “catastrophic illness” under Department of Health guidelines, the patient can submit related information and apply for a catastrophic illness certificate. The application will be formally reviewed, and if approved, the information is entered into his or her IC card. In the present study, the 2000–2011 catastrophic illness registered dataset of Taiwan (including 30 categories) [13] was used to verify the Catastrophic-Illness-Registration (CIR) cases from the sampled population.

In addition, the Charlson comorbidity index (CCI), developed by Charlson and colleagues [14], was calculated to indicate the severity of co-morbidities based on patients’ medical diagnosis codes (ICD-9-CM).

### Statistics

We compared baseline characteristics by descriptive statistics, represented by the numbers of cases, percentages, and means with standard deviation (SD), and cumulative incidence (Inc). The influence of potential confounders on the invasive treatment methods of percutaneous transluminal angioplasty (PTA), bypass surgery and amputation were calculated by Pearson’s chi-square (χ2) test and odds ratios (ORs) with 95% confidence intervals (95% CIs). Significance was set at p = 0.05. Further multivariate analysis by Poisson regression was performed to evaluate the period effect of PTA failure and other potential confounders on the risk of amputation or bypass, as shown by the risk ratio (RR) with the 95% confidence interval (95% CI). All statistical analyses were performed using the Statistical Package for Social Sciences for Windows (SPSS for Windows 19.0).

## Results

**Figure 1:**
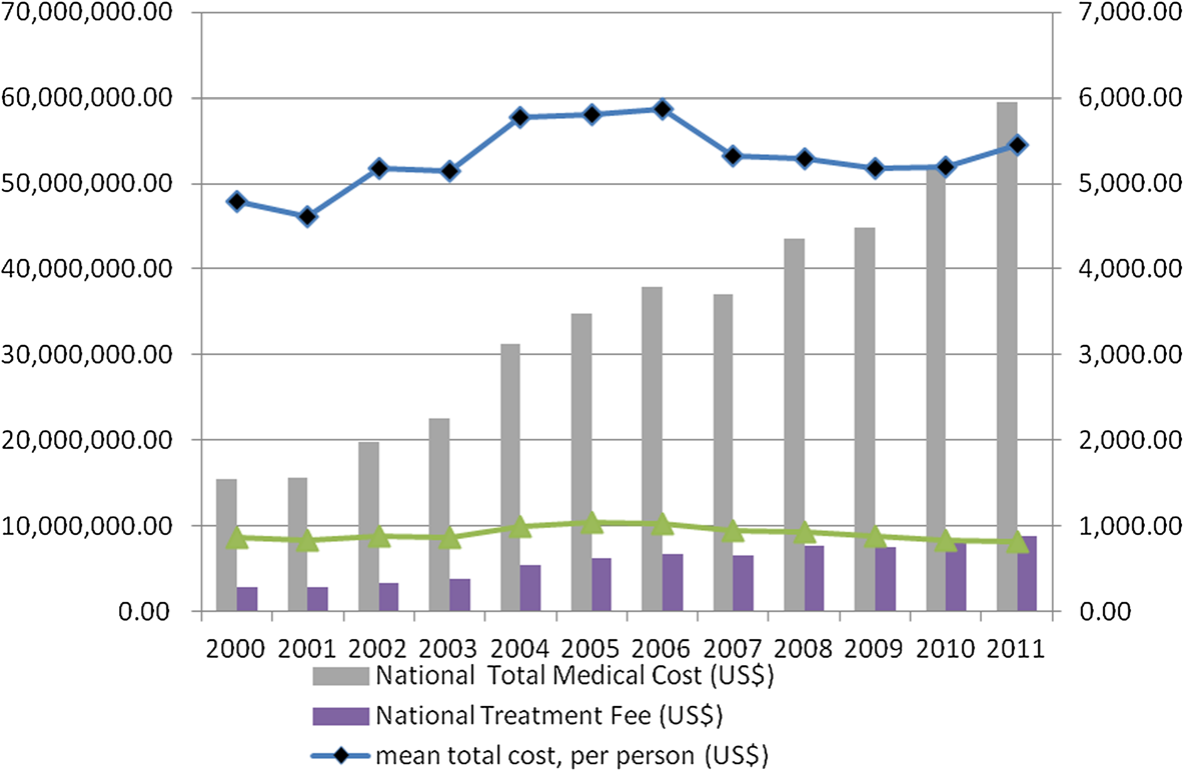
Annual national hospitalization costs and averaged individual hospitalization costs of admitted PAD cases in Taiwan from 2000 to 2011.

Table 2 shows the annual enrolled subjects hospitalized in order to receive one of the invasive treatments for PAD. The invasively-treated incidence of PAD could be estimated classified by gender and age stratum. In general, the invasively-treated incidence of PAD in Taiwan doubled, from 3.73/10,000 (in 2000) to 7.48/10,000 (in 2011). There was an obvious increasing trend of the treated incidence of PAD by age stratum. Male patients were more likely to suffer from PAD with invasive treatment needed, and the incidence ratio (IR) of male *vs* female increased from 1.34 (in 2000, the estimated incidences of the male and female patients being 4.27 and 3.18 per 10,000, respectively) to 1.57 (in 2011, the estimated incidences of the male and female patients being 9.17 and 5.83 per 10,000, respectively). The nationwide and averaged individual medical costs are shown in Figure 1. On average, the total direct medical cost of one hospitalized and invasively-treated PAD case ranged from $US 4,600 to $US 5,900, and the treatment fee accounted for 14.8-18.2% of the total medical cost. Along with the increase in enrolled case numbers annually, the national total medical expenditure for these invasively-treated PAD cases increased greatly from $US 15.5 million per year (in 2000) to $US 59.6 million per year (in 2011) in Taiwan.

**Table 2:** Hospitalized Incidence* of PAD patients receiving any invasive treatment among residents aged 30 years or more in Taiwan, 2000-2011

**Year**	**Total subjects**^**a**^	**Total residents**^**b**^	**Crude incidence**^**c**^	**Male**	**Female**	**30-49.9 years-old**	**50-64.9 years-old**	**65-74.9 years-old**	**75 years-old or more**
				**Inc.**	**Inc.**	**Inc.**	**Inc.**	**Inc.**	**Inc.**
**2000**	**4,446**	**11,917,551**	**3.731**	**4.27**	**3.18**	**0.46**	**3.97**	**13.02**	**21.06**
**2001**	**4,364**	**12,154,222**	**3.591**	**4.06**	**3.11**	**0.45**	**3.87**	**12.72**	**18.60**
**2002**	**4,610**	**12,379,716**	**3.724**	**4.28**	**3.16**	**0.49**	**3.89**	**12.59**	**19.52**
**2003**	**4,778**	**12,598,220**	**3.793**	**4.31**	**3.25**	**0.47**	**3.89**	**12.45**	**19.94**
**2004**	**6,339**	**12,822,685**	**4.944**	**5.76**	**4.12**	**0.62**	**4.85**	**15.91**	**25.85**
**2005**	**7,017**	**13,054,059**	**5.375**	**6.40**	**4.35**	**0.73**	**5.32**	**16.59**	**27.13**
**2006**	**6,773**	**13,362,944**	**5.068**	**6.07**	**4.07**	**0.61**	**4.68**	**15.61**	**26.48**
**2007**	**7,065**	**13,614,777**	**5.189**	**6.29**	**4.10**	**0.68**	**4.84**	**15.86**	**25.96**
**2008**	**7,975**	**13,889,004**	**5.741**	**7.11**	**4.81**	**0.74**	**5.25**	**16.83**	**29.34**
**2009**	**8,437**	**14,182,660**	**5.989**	**7.23**	**4.70**	**0.79**	**5.49**	**17.12**	**29.74**
**2010**	**9,369**	**14,456,222**	**6.481**	**7.84**	**5.16**	**0.83**	**5.56**	**18.99**	**33.35**
**2011**	**11,013**	**14,728,045**	**7.478**	**9.17**	**5.83**	**1.00**	**6.47**	**20.88**	**38.54**

*Incidence (Inc.): 1/10,000

Note: ^a^: This was data-mined from the nationwide health insurance inpatient databank and the cases must meet the inclusion criteria of ICD-9-CM diagnostic codes and ICD-9-CM treatment codes for PAD simultaneously. ^b^: This was selected from the year book of the nationwide population registry; ^c^: This was calculated as (a/b) × 10,000.

**Figure 2:**
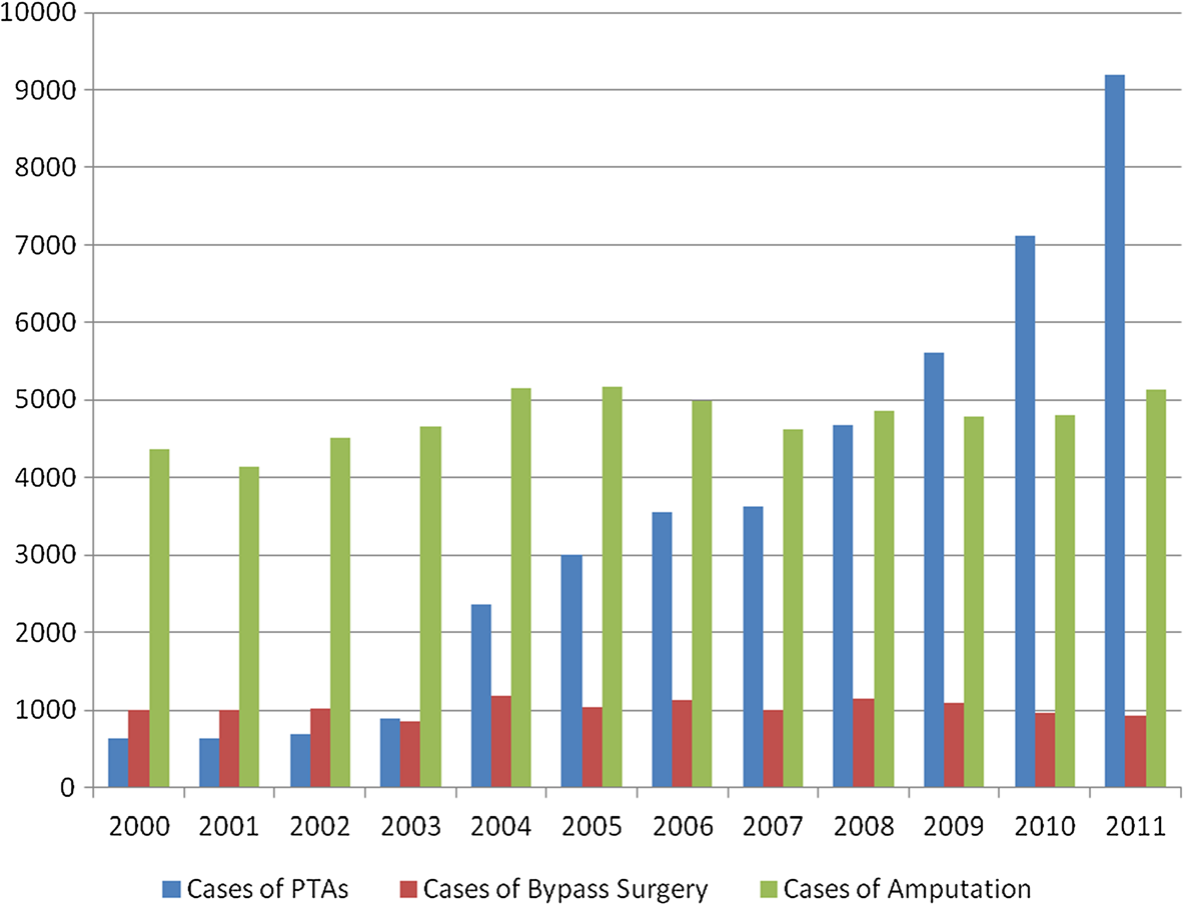
**Annual distributions of various invasive treatment methods for hospitalized PAD cases in Taiwan from 2000 to 2011.** The invasive treatment methods mainly included PTAs, bypass operations or limb amputations.

Figure 2 shows the annual distributions of various invasive treatment methods for hospitalized PAD cases in Taiwan from 2000 to 2011. The invasive treatment methods mainly included PTAs, bypass operations or limb amputations. The annual cases of bypass surgery for the PAD cases averaged 1,000, but the treatment incidence among subjects aged 30 years or more was estimated as 8.4 per 100,000, and reduced to 6.4 per 100,000 from 2000 to 2011. The number of limb amputations varied annually, and ranged from 4,100 to 5,100 during the 12-year study period. However, the amount of PTA procedures remarkably increased by 15 times, from 600/year to 9,100/year from 2000 to 2011. At the same time, the number of hospitals with the ability to perform PTAs doubled from 2000 to 2011. To investigate the possible factors affecting the various invasive treatments for PAD in Taiwan, all three main methods to treat PAD invasively were summed from 2000 to 2001. Table 3 shows the age, gender, co-morbidity and socio-economic status effects on the performance of different invasive treatment methods for the hospitalized PAD cases in Taiwan. In total, 51.3% of the enrolled cases were treated with limb amputation, and female, young and middle-aged people (30–50 and 50–65 years of age), DM patients and those on a low income had a tendency to undergo amputation due to PAD (significant odds ratios (ORs): 1.12, 1.30, 11.22, 6.12 and 1.41, respectively). In total, 37.6% of the enrolled cases were treated with PTAs; hypertension, cardiovascular disease, and hyperlipidemia patients, and people with Catastrophic Illness Registration (CIR) were at greater risk of receiving a PTA procedure for PAD (significant OR: 3.11, 3.45, 7.20 and 1.81, respectively). Only ESRD patients were more likely to receive a vascular surgery (significant OR: 1.36).

**Table 3:** Summed various invasive treatment methods for hospitalized PAD cases and associated factors in Taiwan from 2000–2011

	**Total cases (%) 111,495**	**PTA (n = 41,987, 37.6%)**		**Bypass (n = 12,334, 11.1%)**		**Amputation (n = 57,174, 51.3%)**		**p-value (χ**^**2 **^**test)**	** Crude OR (95% CI)**
		**No.**	**(%)**	**No.**	**(%)**	**No.**	**(%)**		
**Gender**									
**Male**	**60,187 (59.5%)**	**25,372**	**60.4**	**7,772**	**63.0**	**33,366**	**58.4**	**<0.001**	
**Female**	**40,936 (40.5%)**	**16,615**	**39.6**	**4,562**	**37.0**	**23,808**	**41.6**^**a**^		**1.12**^**a **^**(1.09-1.14)**
**Age Stratum**									
**30–49.9 y/o**	**7,063 (7.0%)**	**2,594**	**6.2**	**748**	**6.1**	**4,182**	**7.3**^**a**^	**<0.001**	**1.30**^**a **^**(1. 24–1.37)**
**50–64.9 y/o**	**26,138 (25.8%)**	**10,405**	**24.8**	**2,704**	**21.9**	**15,317**	**26.8**^**a**^		**1. 22**^**a **^**(1.18-1.25)**
**65–74.9 y/o**	**31,070 (30.7%)**	**12,820**	**30.5**	**4,107**	**33.3**^**b**^	**17,537**	**30.7**		**1.03**^**b **^**(0.98-1.08)**
**> = 75y/o**	**36,866 (36.5%)**	**16,169**	**38.5**	**4,775**	**38.7**	**20,138**	**35.2**		
**Diabetes**									
**Yes**	**69,026 (68.2%)**	**20,282**	**48.3**	**6,978**	**56.6**	**48,318**	**84.5**^**a**^	**<0.001**	**6.12**^**a **^**(5.95-6.31)**
**Hypertension**									
**Yes**	**45,961 (45.4%)**	**25,844**	**61.5**^**c**^	**5,918**	**48.0**	**18,635**	**32.6**	**<0.001**	**3.11**^**c **^**(3.03-3.19)**
**ESRD**									
**Yes**	**11,318 (11.2%)**	**4,094**	**9.7**	**1,741**	**14.1**^**b**^	**6,313**	**11.0**	**<0.001**	**1.36**^**b **^**(1.29-1.44)**
**CAD**									
**Yes**	**16,072 (15.9%)**	**10,727**	**25.5**^**c**^	**2,254**	**18.3**	**4,635**	**8.1**	**<0.001**	**3.45**^**c **^**(3.33-3.58)**
**Dyslipidemia**									
**Yes**	**4,803 (4.7%)**	**3,960**	**9.4**^**c**^	**438**	**3.6**	**690**	**1.2**	**<0.001**	**7.20**^**c **^**(6.68-7.77)**
**Group identified**									
**Normal**	**72,520 (71.7%)**	**27,500**	**65.5**	**9,530**	**77.3**	**43,391**	**75.9**	**<0.001**	
**Low-income**	**2,704 (2.7%)**	**875**	**2.1**	**249**	**2.0**	**1,831**	**3.2**^**a**^		**1.41**^**a **^**(1.30-1.53)**
**CIR**	**25,913 (25.6%)**	**13,620**	**32.4**^**c**^	**2,557**	**20.7**	**11,957**	**20.9**		**1.81**^**c **^**(1.76-1.87)**

OR: odds ratio calculated by the Chi-square method; CI: Confidence Interval.

ESRD: end-stage renal disease; CAD: cardiovascular disease.

CIR: Catastrophic Illness Registration.

Note: ^a^: This represents the odds ratio of amputation in this subgroup compared to the others. ^b^: This represents the odds ratio of bypass surgery in this subgroup compared to the others. ^c^: This represents the odds ratio of PTAs in this subgroup compared to the others.

**Figure 3:**
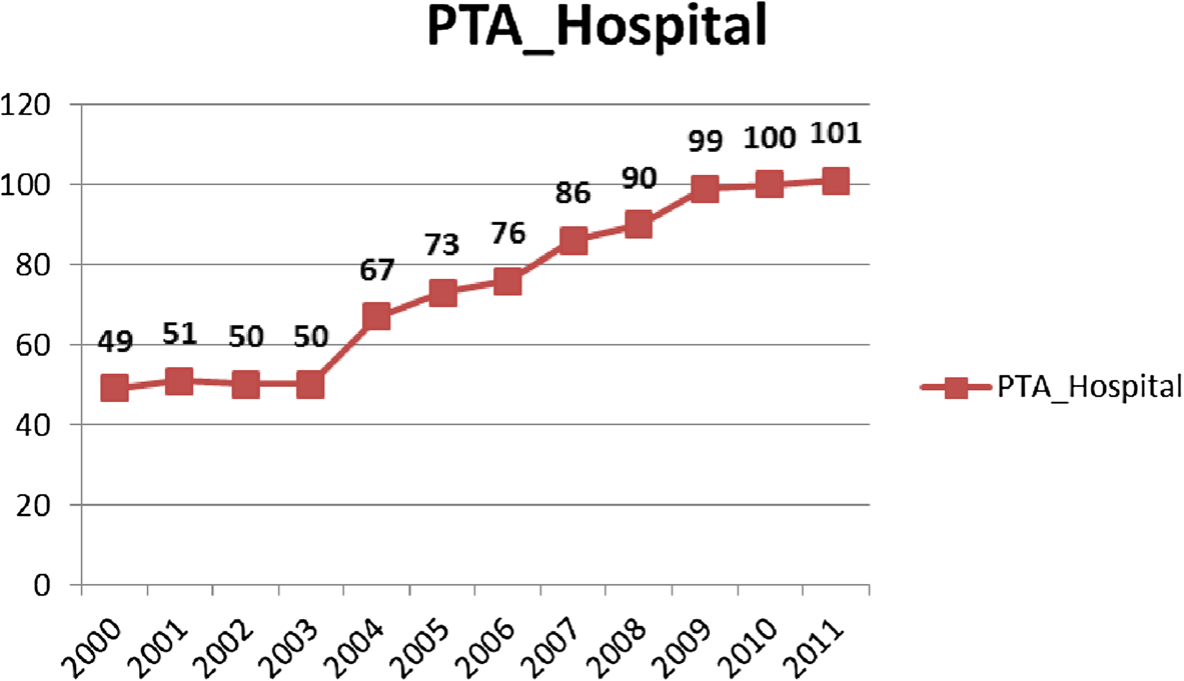
The annual numbers of hospitals with the ability to perform PTA procedures in Taiwan, 2000–2011.

Interestingly, a co-linear relationship between PTA cases and hospitals with the ability to perform PTAs during different periods (2000–2003 and 2004–2011) in Taiwan was found when comparing Figure 2 and Figure 3. Therefore, the outcome of these dominantly increased PTA cases is worthy of evaluation. Three cohort subgroups (2000–2001 cohort, 2004–2005 cohort and 2008–2009 cohort) were selected in order to evaluate their middle-term (two years) results after PTA treatment (Table 4). To calculate the failure rate of PTAs, all cases referred for another bypass surgery or amputation procedure were recorded, and there were 22.13% (7.27% referred for a bypass; 14.86% referred for an amputation), 11.91% (3.75% and 8.16% referred for a bypass and amputation, respectively) and 10.61% (2.10% and 8.51% referred for a bypass and amputation, respectively) failure rates of original PTAs among the first (2000–2001), second (2004–2005) and third (2008–2009) cohorts, respectively. A declining period effect on PTA failure could be identified. Poisson regression models were used to evaluate the associated factors related to failed PTAs among the three cohort groups. In the first cohort, aging was the most dominant factor (significant relative risks (RR) ranging from 3.5 to 5.0 compared with the 30–50 years of age group); in the second cohort, the female gender and higher co-morbidities were major factors (significant RR: 1.2 and 1.3, respectively). Regarding the most recent (third) cohort, there was an additive effect of co-morbidities and a negative effect of low-income status on the failure of original PTAs (significant RR: 1.5 and 0.6, respectively).

**Table 4:** **Poisson regression model of PTA failure**^**a **^**in the PAD cases for various time periods in Taiwan**

	**Cohort I: PTAs performed during 2000-2001**		**Cohort II: PTAs performed during 2004-2005**		**Cohort III: PTAs performed during 2008-2009**	
***Descriptive data for both failed patterns***	**No.**	**(%)**	**No.**	**(%)**	**No.**	**(%)**
**Enrolled cases**	**1,265**		**5,356**		**10,258**	
**Referred for bypass within 2 years**	**92**	**7.27%**	**201**	**3.75%**	**215**	**2.10%**
** Referred for amputation within 2 years**	**188**	**14.86%**	**437**	**8.16%**	**873**	**8.51%**
***Period effect***^***b ***^ ***of PTAs Failure (RR, 95% C.I.)***	***1.00***		***0.92***	***0.80-1.04***	***0.92***	***0.80-1.06***
***Analytic data for all PTA failed cases***	**RR**	**(95% CI)**	**RR**	**(95% CI)**	**RR**	**(95% CI)**
**Age Stratum**						
***30-49.9 y/o***	**1.00**		**1.00**		**1.00**	
***50-64.9 y/o***	**3.54**	**1.68 ~ 5.49**	**1.45**	**0.84 ~ 1.85**	**1.10**	**0.73 ~ 1.42**
***65-74.9 y/o***	**4.95**	**2.41 ~ 10.13**	**1.54**	**0.82 ~ 1.76**	**1.17**	**0.78 ~ 1.50**
***75 y/o or more***	**4.56**	**2.22 ~ 9.36**	**1.63**	**0.83 ~ 1.79**	**0.92**	**0.72 ~ 1.19**
**Gender**						
***Male vs female***	**0.87**	**0.69 ~ 1.11**	**0.85**	**0.72-0.99**	**1.10**	**0.97 ~ 1.26**
**Co-morbidities (CCI Index)**						
***Higher (≥4) vs lower***	**1.54**	**0.95 ~ 2.43**	**1.31**	**1.02 ~ 1.67**	**1.46**	**1.20 ~ 1.77**
**Socioeconomic level**						
***Low-income vs other***	**1.22**	**0.39 ~ 3.82**	**1.39**	**0.74 ~ 2.59**	**0.58**	**0.34 ~ 0.99**

RR: risk ratio.

Note: ^a^: This means that all the patients needed to be treated with additive bypass surgery or amputation within 2 years after their PTA procedure. ^b^: Crude relative ratio of period effect is to compare with the PTAs failure rate in period I and to keep off age-period interaction.

## Discussion

PAD is common, and much research has been conducted to identify possible risk factors for PAD [15-17], evaluate appropriate diagnostic modalities for PAD [18], and develop effective treatments for symptomatic PAD [19,20]. LE PAD is a common vascular condition that affects both quality of life and life expectancy, with an increased risk of cardiovascular events. Choice of endovascular or surgical intervention remains controversial in an ever-evolving field, and tissue engineering is a developing area and aims to produce grafts with similar patency [6]. The exact incidence or prevalence of LE PAD is difficult to be evaluated due to different people, different nations, and even different states of one country. For example, a multi-centers study at 350 primary care practices of 25 cities in the United States, enrolling a total of 6979 PAD patients, showed that among them, the clinical presentation varies from no symptoms to atypical rest pain, intermittent claudication, ischemic ulcers, or gangrene. Incidence of PAD throughout the US was various from 10% to 29%, with or without symptoms [21]. In Japanese patients with PAD, women were found to have more severe symptomatic states and uncontrolled risk factors, and the prevalence of iliac artery lesions was lower, but below the knee lesions were more severe in women [22]. Rapid progression of PAD was found in hemodialysis Taiwanese patients, and the prevalence of ABI <0.9 increased yearly (10.4%, 22.7% and 27.9%, respectively; p < 0.001) [23]. Another retrospective study of a Singapore hospital discharge database (2004–2009) noted that DM patients with renal disease had significantly higher rates of lower extremity amputation (7.1%) compared to DM patients without renal disease (2.5%, p < 0.001) [24]. In the present study, gender, various age strata and co-morbidity (including diabetes, hypertension, ESRD, cardiovascular disease (CAD), hyperlipidemia and an integrated co-morbidity index) were found to have significant effects on the performance of different invasive treatment methods for hospitalized PAD cases in Taiwan.

Some epidemiological studies of asymptomatic PAD in Taiwan have been performed in specific disease groups. The ABI was similarly used to detect PAD (ABI < 0.90). For example, the records of 484 Taiwanese patients with end-stage renal disease (ESRD) were reviewed and PAD had an overall prevalence of 18.2% and was significantly more common in hemodialysis (HD) patients (21.8%) than in peritoneal dialysis (PD) patients (4.8%) [25]. Another prospective cross-sectional study showed that the prevalence of asymptomatic PAD among COPD patients in Taiwan is lower (2.5% in the younger participants (<65 years of age, n  =  118) and 10% in the elderly participants (≥65 years of age, n  =  309)) than in Western countries [26]. For the general Taiwanese population, a recent survey enrolling ambulatory participants without symptoms of PAD revealed that the overall prevalence of asymptomatic PAD was 5.4% (2.8% in the younger participants [<65 years of age, n = 1021] and 8.4% in the elderly participants [≥65 years of age, n = 894]) [27]. The present study showed an invasive treatment incidence of LE PAD in Taiwan and the latest cumulative incidence of 7.48 per 10,000 in general population was estimated in 2011. Age and period effects had been noted. Otherwise, a gender difference was also observed, and the incidence ratio (IR) of male *vs* female increased from 1.34 (in 2000) to 1.57 (in 2011). A significant increase in the PAD prevalence with age has also been noted in American adults, the PAD prevalence being 12.2% (95% confidence interval (CI) = 10.9-13.5%); 7.0% (95% CI = 5.6-8.4%) for those aged 60 to 69; and 12.5% (95% CI = 10.4-14.6%) and 23.2% (95% CI = 19.8-26.7%) for those aged 70 to 79 and 80 and older [28].

The economic burden of PAD is high. Among the US Medicare population, Medicare program outlays totaled $3.87 billion for PAD and 88% of expenditures were for inpatient care. In total, 6.8% of the elderly Medicare population received treatment for PAD. Treatment increased with age, with rates of 4.5%, 7.5%, and 11.8% for individuals aged 65–74, 75–84, and >85 years, respectively [29]. In the present study, the national total medical expenditure for these invasively-treated PAD cases was found to have increased quickly, from $US 15.5 million per year (in 2000) to $US 59.6 million per year (in 2011). Besides, the hospitalization incidence of PAD was found to be at least 20 times higher in the elderly (65 years or greater) than in the young (<50 years). This indicated an increased medical burden of LE PAD, and much more care for aged people should be instigated by the health policy authority in Taiwan. A study based on the REduction of Atherothrombosis for Continued Health (REACH) Registry to estimate the 2-year associated costs in US patients with established PAD showed that the mean cumulative hospitalization costs per patient were $7,445, $7,000, $10,430, and $11,693 for patients with asymptomatic PAD, a history of claudication, lower-limb amputation, and revascularization, respectively (p = 0.007) [30]. In Taiwan, the present study showed that the total direct medical cost of one hospitalized and invasively-treated PAD case ranged from $US 4,600 to $US 5,900 on average, which is much lower than other countries.

There is now a trend towards endovascular revascularization for most PAD patients. A study using the US Nationwide Inpatient Sample (NIS) database (1999–2007), identifying patients who had an identifiable ICD-9 diagnosis code of atherosclerotic disease (claudication [440.21] or limb threat [440.22-440.24]), showed that the number of patients per year undergoing PTA increased threefold [31]. Much more dramatically in Taiwan, the present study revealed that the number of PTA procedures remarkably increased by 15 times from 2000 to 2011, which perhaps was partially contributed to by the doubled numbers of hospitals with the ability to perform PTA skills from 2000 to 2011. Data of US Medicare beneficiaries analyzed between 1996 and 2006 revealed that bypass surgery decreased by 42% (219 to 126 per 100,000; RR = 0.58; 95% CI: 0.5-0.7) [32]. In Taiwan, the incidence of bypass surgery for PAD cases among residents aged 30 years or more was estimated as 8.4 per 100,000 and reduced to 6.4 per 100,000 from 2000 to 2011 in the present study, which showed a similar decreasing trend but was much lower than the study mentioned above. The deployment of medical resources for vascular surgeries in Taiwan may be further evaluated by the health policy authority.

Traditionally, amputation represents end-stage failure for those with LE PAD. Using data from the US Centers for Medicare & Medicaid Services (CMS) from 2000 to 2008, among 2,730,742 older patients (aged 65 years or more) with identified PAD, the overall rate of LE amputation decreased from 7,258 per 100,000 patients with PAD to 5,790 per 100,000 (p < 0.001 for trend) [33]. In the present study, the number of limb amputations varied annually, and ranged between 4,100 and 5,100 per year (equal to 34 to 40 per 100,000) for the general population. Compared to the above study in the US, the amputation rate for LE PAD patients in Taiwan was lower. Another study using the US Nationwide Inpatient Sample (NIS) database (1999–2007) revealed that in-hospital amputation rates were significantly higher for patients who had PTA (7%) than a peripheral bypass graft (BPG) (3.9%, odds ratio [OR], 1.67 [1.49-1.85]; p < 0.01) or patients who underwent aorto-femoral bypass (ABF) (3.0%; OR, 2.32 [1.79, 3.03]; p < 0.01) [31]. In our present study, we calculated the 2-year failure rate of PTAs, and found these to be 22.13%, 11.91% and 10.61% (including 14.86%, 8.16% and 8.51% referred for amputation) among the first (2000–2001), second (2004–2005) and third (2008–2009) cohorts, respectively. Otherwise, aging, the female gender and higher co-morbidities were found to be associated with the above 2-year PTA failure rates.

Socioeconomic disparities could persist in the amputation rates of LE PAD. Data from the US Nationwide Inpatient Sample (NIS) from 1998 to 2002 showed that multivariate analysis indicated significantly higher odds of amputation associated with the following variables: nonwhites (1.91, 95% confidence interval [CI], 1.65, 2.20), low-income bracket (1.41, 95% CI, 1.18, 1.60), and Medicare & Medicaid patients (1.81, 95% CI, 1.66, 1.97) [34]. Another study of NIS data comparing two periods (2001–2003 and 2004–2007) found that annually, the total number of interventions increased by 15% and the average annual number of endovascular interventions increased by 78% (p < 0.001). After adjusting for age and co-morbidities, African Americans were found to have a 2.4 times greater odds of amputation as compared with Caucasians, whereas those under Medicare or Medicaid had a 1.5 times greater odds [35]. These economically disadvantaged patients were thought to have had a delayed diagnosis of peripheral vascular disease, probably due to lack of adequate primary care or access to vascular interventions, or both. In Taiwan, the policy of listing NHI-defined catastrophic illnesses exempts some vulnerable populations from the co-payment economic burden and protects their human rights with regards to access to necessary medical care. A recent study revealed that the prevalence of certificated catastrophic illness in Taiwan’s elderly population utilizing ambulatory medical services was 10.16%. On average, 61.62 emergency department (ED) visits/1,000 persons (95% CI: 59.22–64.01) per month was estimated for elderly Taiwanese with a catastrophic illness, which was significantly greater than that for the elderly without a catastrophic illness (mean 33.53, 95% CI: 32.34–34.71). A significantly greater total medical expenditure for emergency care was observed in the catastrophic illness subgroup ($US 145.6 ± 193.5) as compared with the non-catastrophic illness group ($US 108.7 ± 338.0) (*p* < 0.001) [13]. In the present study, these disadvantaged populations, including low-income people and catastrophic illness certificated patients, were evaluated. Among 51.3% of all the enrolled PAD cases treated with limb amputations, low-income people had a tendency to undergo amputation due to their condition (OR: 1.41, 95% CI: 1.30-1.53); 37.6% of those treated with PTAs with CIR had a greater opportunity to receive a PTA procedure (OR: 1.81, 95% CI: 1.76-1.87). Perhaps there was a higher opportunity to receive amputation for those on a low income, and a negative effect of low-income status on the failure of original PTAs could be observed.

## Conclusions

In Taiwan, a gender difference and age and period effects on the invasively-treated incidence of LE PAD were observed, and the total direct medical cost of one hospitalized and invasively-treated PAD case was relatively cheap. Female, young and middle-aged people (30–50 and 50–65 years of age), DM patients and those on a low income had a tendency to undergo amputation due to PAD. The number of PTA procedures remarkably increased, partially contributed to by the number of hospitals performing PTAs having doubled, and the 2-year failure rate of PTAs reduced from 2000 to 2011.

## Competing interest

All authors declare that they have no conflicts of interest, including directorships, stock holding or contracts.

## Authors’ contributions

The study was designed by NPY, NTC and CLC; data were gathered and analyzed by YTL, JCH and YNH; the initial draft of the manuscript was written by NPY, DC and NTC; the accuracy of the data and analyses was assured by CLC and DC; and the draft was revised critically by NTC and NPY. All authors participated in the preparation of the manuscript and approved the final version. All authors read and approved the final manuscript.

## Pre-publication history

The pre-publication history for this paper can be accessed here:

http://www.biomedcentral.com/1471-2458/13/1107/prepub
